# CTCF as a regulator of alternative splicing: new tricks for an old player

**DOI:** 10.1093/nar/gkab520

**Published:** 2021-06-28

**Authors:** Adel B Alharbi, Ulf Schmitz, Charles G Bailey, John E J Rasko

**Affiliations:** Gene & Stem Cell Therapy Program Centenary Institute, The University of Sydney, Camperdown, NSW 2050, Australia; Department of Laboratory Medicine, Faculty of Applied Medical Sciences, Umm Al-Qura University, Makkah, Saudi Arabia; Computational BioMedicine Laboratory Centenary Institute, The University of Sydney, Camperdown, NSW 2050, Australia; Faculty of Medicine & Health, The University of Sydney, NSW 2006, Australia; Cancer & Gene Regulation Laboratory Centenary Institute, The University of Sydney, Camperdown, NSW 2050, Australia; Gene & Stem Cell Therapy Program Centenary Institute, The University of Sydney, Camperdown, NSW 2050, Australia; Computational BioMedicine Laboratory Centenary Institute, The University of Sydney, Camperdown, NSW 2050, Australia; Faculty of Medicine & Health, The University of Sydney, NSW 2006, Australia; Gene & Stem Cell Therapy Program Centenary Institute, The University of Sydney, Camperdown, NSW 2050, Australia; Faculty of Medicine & Health, The University of Sydney, NSW 2006, Australia; Cancer & Gene Regulation Laboratory Centenary Institute, The University of Sydney, Camperdown, NSW 2050, Australia; Gene & Stem Cell Therapy Program Centenary Institute, The University of Sydney, Camperdown, NSW 2050, Australia; Faculty of Medicine & Health, The University of Sydney, NSW 2006, Australia; Cell & Molecular Therapies, Royal Prince Alfred Hospital, Camperdown, NSW 2050, Australia

## Abstract

Three decades of research have established the CCCTC-binding factor (CTCF) as a ubiquitously expressed chromatin organizing factor and master regulator of gene expression. A new role for CTCF as a regulator of alternative splicing (AS) has now emerged. CTCF has been directly and indirectly linked to the modulation of AS at the individual transcript and at the transcriptome-wide level. The emerging role of CTCF-mediated regulation of AS involves diverse mechanisms; including transcriptional elongation, DNA methylation, chromatin architecture, histone modifications, and regulation of splicing factor expression and assembly. CTCF thereby appears to not only co-ordinate gene expression regulation but contributes to the modulation of transcriptomic complexity. In this review, we highlight previous discoveries regarding the role of CTCF in AS. In addition, we summarize detailed mechanisms by which CTCF mediates AS regulation. We propose opportunities for further research designed to examine the possible fate of CTCF-mediated alternatively spliced genes and associated biological consequences. CTCF has been widely acknowledged as the ‘master weaver of the genome’. Given its multiple connections, further characterization of CTCF’s emerging role in splicing regulation might extend its functional repertoire towards a ‘conductor of the splicing orchestra’.

## INTRODUCTION

### CTCF – an old player

CCCTC-binding factor (CTCF) was first identified as a nuclear DNA-binding protein, which negatively regulates chicken *c-myc* expression by interacting with three regularly spaced repeats of the CCCTC DNA motif (1). Since then, considerable attention has been focused on CTCF leading to pivotal discoveries related to its structure, binding activities and functions. CTCF is a multivalent 11-zinc finger DNA-binding protein that is ubiquitously expressed in most tissues of vertebrate species (1–4). Combinatorial usage of CTCF zinc fingers (4,5) allows its binding to tens of thousands of conserved DNA sites as revealed by chromatin immunoprecipitation sequencing (ChIP-seq) experiments (6–8). More than 30% and 50% of these binding sites are located in intronic and intergenic regions, respectively. These sites are mostly ubiquitous; however, cell-type-specific CTCF binding is observed (6–8). Ubiquitous CTCF target sites are predominantly localized within intergenic regions and are highly conserved compared to cell-type-specific ones, which are mostly located within introns (8).

CTCF has also been characterized as an RNA-binding protein and these interactions are essential for facilitating CTCF-mediated chromatin architecture (9–13). Such interactions are involved in some CTCF activities including CTCF dimerization, distal genomic binding sites, chromatin looping and gene regulation (9–12). Indeed, locus- or transcript-specific functions for CTCF-RNA interactions have been clearly demonstrated (9,13,14). CTCF can also undergo multiple post-translational modifications which could affect interactions with binding partners. These interactors include transcription factors, chromatin remodelers, methylation regulators, histone modifiers and splicing factors (reviewed in (15–17)).

The complexity of CTCF interactions is further expanded by the ability of CTCF to homodimerize and mediate contacts with multiple distal intra- and inter-chromosomal target sites across the genome. Such interactions can facilitate genome folding into topologically associating domains (TADs) and chromatin loops, encompassing those interaction domains (18–21). This allows CTCF to coordinate chromatin and genome architecture, which reinforce CTCF’s eminence as ‘the master weaver of the genome’ (22). Chromatin looping occurs when a DNA-bound CTCF molecule interacts with another CTCF molecule located at a proximal or distal genomic site (21,23). These CTCF–CTCF interactions mostly initiate chromatin loops when the CTCF-bound sites are in a convergent (i.e. forward and reverse) orientation (23,24) rather than divergent orientation, which has been mostly observed at non-looped TAD boundaries (20,24). CTCF also co-ordinates chromatin architecture in concert with the cohesin complex to help define and stabilize chromatin loops. While CTCF associates with over half of the cohesin binding sites genome-wide, both CTCF and cohesin also have their own specific binding sites and independent roles in coordinating chromatin organization (25–28).

Given its unique role in higher-order chromatin architecture and the multiplicity and specificity of its binding sites, CTCF is able to regulate diverse molecular and epigenetic functions, summarized herein. CTCF is a unique regulator of gene expression, which can function as a transcriptional repressor and activator as well as a chromatin insulator, the latter occurring through interference of the contact between enhancers/silencers and promoters (5,22). Furthermore, it epigenetically regulates gene expression through gene imprinting, X-chromosome inactivation, and by preserving methylation-free regions throughout the genome (15,29). Moreover, CTCF-mediated chromatin looping modulates gene expression by bringing genomic loci into spatial proximity (30–32).

Compelling evidence has linked CTCF to modulation of alternative splicing (AS) at both the individual transcript and transcriptome-wide level (33–46). AS is a complex biological process, which affects over 95% of human multi-exonic genes and enriches transcriptome and protein diversity by facilitating the production of multiple mRNA and protein isoforms from individual genes (47,48). There are four major forms of AS including exon skipping, mutually exclusive exons, alternative use of 5′ or 3′ splice sites and intron retention. With more than 40% prevalence, the most common form of AS is exon skipping, which involves the exclusion of one or more exons from the mature mRNA transcript. Splicing can occur at a site alternative to the canonical splice site, depending on whether the new splice site replaces the canonical donor or acceptor site. Mutually exclusive exons are exons from the same gene that can be differentially included in the spliced gene transcript, i.e. they are rarely found together in the same mature mRNA. Finally, intron retention is a process by which an intron is not excised from the pre-mRNA and therefore retained in the mature mRNA transcript, often leading to nonsense-mediated mRNA decay (47,48).

CTCF’s involvement with AS, identified over the past decade, has expanded the list of mechanisms by which CTCF can regulate gene expression and transcriptomic complexity (33–46); however, its direct impact on biological functions has not been fully elucidated. Herein, we highlight the CTCF-mediated mechanisms linked to AS regulation. While the majority of these mechanisms have not been fully characterized, we emphasize the extent to which CTCF has been experimentally verified as a regulator of AS. Furthermore, we track the putative roles of CTCF in modulating AS, placing them in context of the regulatory mechanisms governing the transcriptional and splicing machinery. This review provides a roadmap toward understanding and further studying the under-recognized role of CTCF in AS regulation.

## NEW TRICKS: CTCF IN ALTERNATIVE SPLICING REGULATION

**Figure 1.**
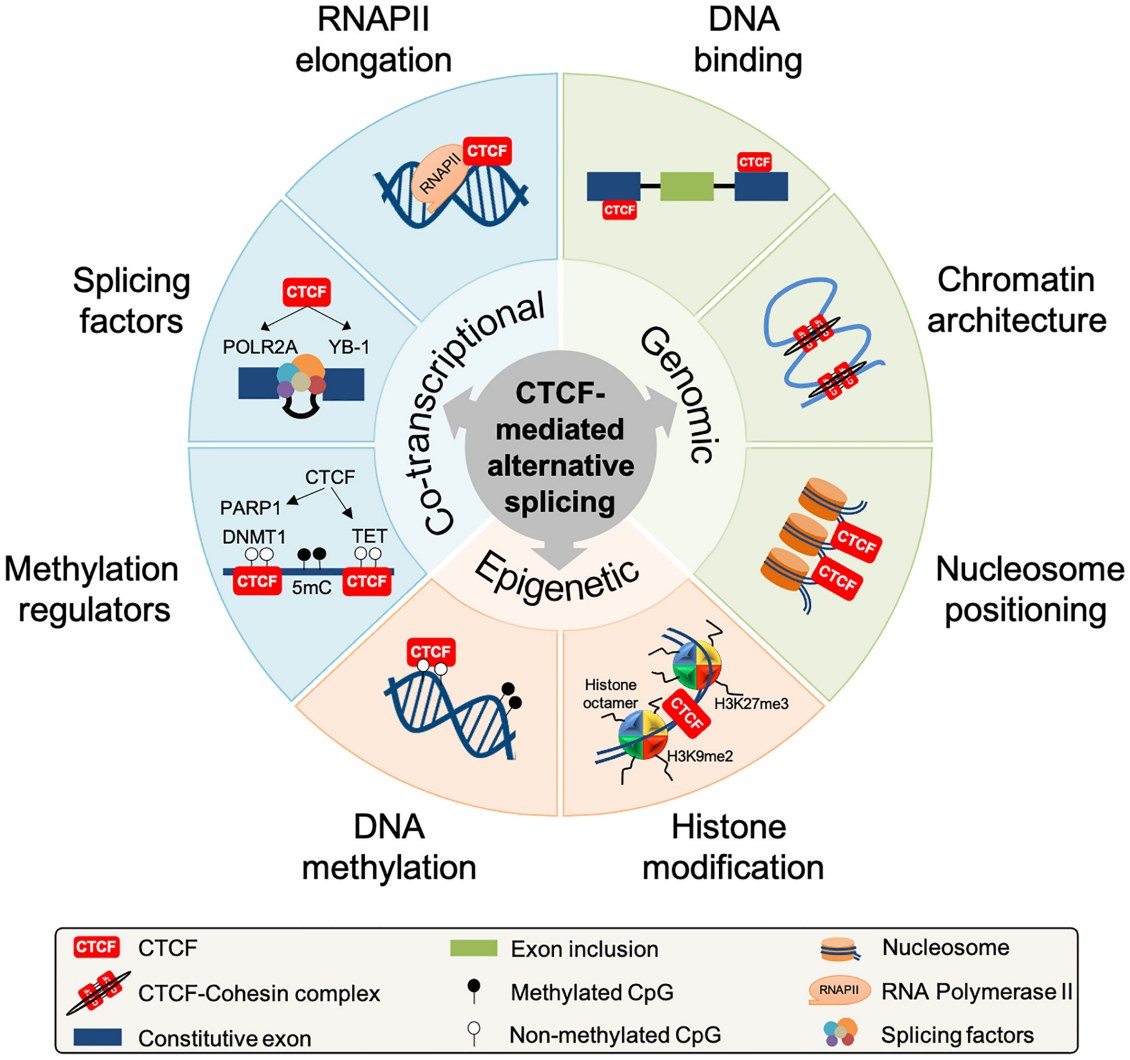
Mechanisms of CTCF-mediated regulation of alternative splicing. CTCF has been linked to key determinants of AS regulation which can be broadly categorized into co-transcriptional, genomic and epigenetic mechanisms. During co-transcriptional regulation, CTCF-mediated AS is regulated by stalling RNAPII transcriptional elongation (33–35,42), controlling DNA methylation (60–62) and recruitment of splicing factors (42,58,66). While the co-transcriptional factors have been mostly experimentally verified, the genomic and epigenetic regulatory roles of CTCF in modulating AS are putative. These involve localization of CTCF binding sites proximal to splice sites (33–38,40–42,44), chromatin architecture (35,36), nucleosome enrichment (7,85–87), histone modification (35,46) and regulation of CTCF binding via DNA methylation patterns (33,34,36,38,40,41).

It is widely accepted that AS re-configures the transcriptome and proteome to facilitate certain biological processes. Perturbations to this highly calibrated process can cause diseases such as Hutchinson-Gilford Progeria syndrome, Duchenne muscular dystrophy, melanoma and breast cancer (49,50). Thus, maintaining tight control of AS requires a complex regulatory network integrating co-transcriptional, genomic and epigenetic factors such as transcriptional elongation, DNA methylation, chromatin architecture, histone modifications, and splicing factor expression and assembly (51,52). Over the past decade, CTCF has been characterized as a direct and indirect modulator of AS decisions. This function is achieved through various mechanisms involving multiple factors including co-transcriptional regulation, genomic features and epigenetic factors (33–46), as summarized (Figure 1) and discussed henceforth.

## CO-TRANSCRIPTIONAL REGULATION

### RNA polymerase II elongation and archetypal *CD45* locus

**Figure 2.**
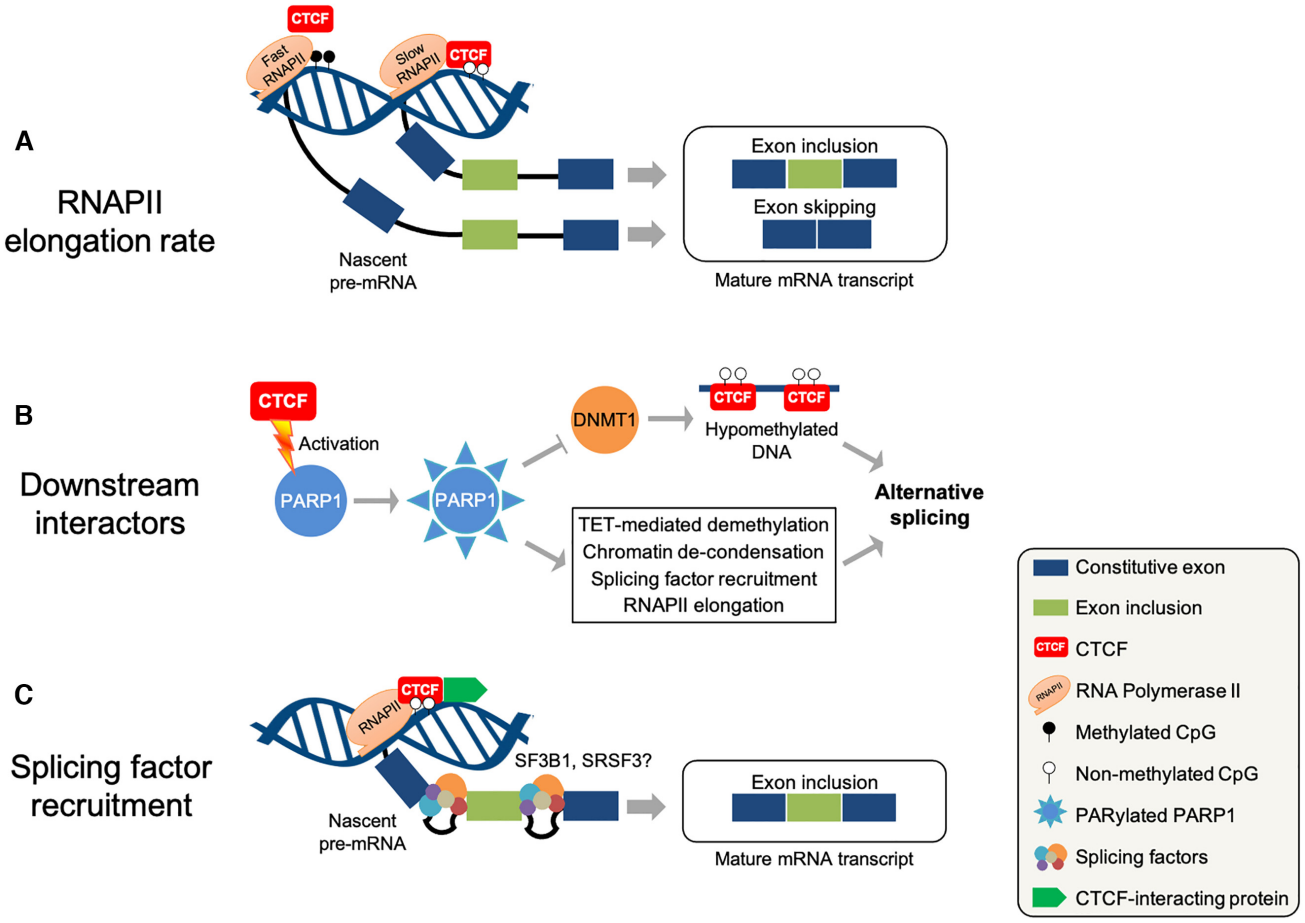
CTCF-mediated co-transcriptional regulation of alternative splicing. Schematic representation of co-transcriptional mechanisms by which CTCF modulates AS. (**A**) The ‘roadblock’ mechanism involves altering RNAPII elongation rate in a methylation-dependent manner (33,34). (**B**) CTCF regulation of DNA methylation via activation of PARP1 and PARylation (60,61), which are also involved in other AS-related regulatory activities (66–69). (**C**) Splicing factor recruitment might also take part in CTCF-mediated AS regulation through direct interaction with RNA binding proteins (42,58,70) or other transcription factors involved in AS such as PARP1 (66), MeCP2 (38), YB-1 (108,109) and HP1α (46,124,127). SF3B1 and SRSF3 are examples of RNA-binding proteins.

RNA polymerase II (RNAPII) elongation is fundamental to transcription and constitutive splicing processes, which require an optimal RNAPII elongation rate for producing canonical mature mRNA transcripts. This means that slowing or accelerating transcription elongation can both inhibit or enhance splice site recognition depending on additional factors. Alteration of the elongation rate influences inclusion of exons or introns particularly those having weak splice sites and short flanking introns (53–56). In a pivotal discovery, Shukla *et al.* revealed the first direct link ‘roadblock model’ between CTCF and AS regulation involving RNAPII elongation and methylation of CTCF binding sites. Binding of CTCF to *CD45* exon 5 impedes RNAPII elongation which allows the spliceosome to assemble at the weak upstream splice site and eventually leads to the inclusion of exon 5 in human Burkitt lymphoma B cells (33) (Figure 2A). AS of *CD45* exons 4, 5 and 6 leads to the generation of multiple isoforms, which have been tightly linked to lymphocyte development (57). This putative new role for CTCF in the regulation of lymphocyte development demands further study.

The decision for CTCF-mediated *CD45* exon 5 inclusion is tightly regulated by the methylation status of a CTCF binding site in *cis*. CTCF depletion or loss of CTCF binding due to methylation within its cognate binding site supports faster RNAPII elongation and subsequently exon exclusion (33,34). While 5‐methylcytosine (5mC) evicts CTCF and promotes exon exclusion, DNMT1 depletion decreases 5mC levels at exon 5 and promotes CTCF binding and exon inclusion (33). In addition, members of the TET (Ten-eleven translocation) family of methylcytosine dioxygenases (TET1 and TET2) oxidize 5mC to 5-hydroxymethylcytosine (5hmC) and its downstream oxidative intermediates. This can subsequently promote CTCF binding and exon inclusion, which is reversed by depleting TET1 and TET2 expression (34).

In line with this context‐dependent regulatory mechanism, a global correlation between CTCF, RNAPII occupancy and exon inclusion was detected when CTCF binding occurs within ∼1 kb downstream of alternatively spliced exons in BJAB and BL41 cells (33). This has been independently supported by a stronger association between CTCF and RNAPII sites mostly downstream (∼0.6 kb) of differentially included exons in MCF7 breast cancer cells compared to non-tumorigenic MCF10A mammary cells (46). Furthermore, there were global reciprocal exchanges of 5mC and 5hmC at CTCF binding sites downstream (∼1.5 kb) of differentially alternatively spliced exons in naive and activated CD4^+^ T cells, where 5mC and 5hmC favor upstream exon exclusion and inclusion, respectively. Nevertheless, the highest enrichment of CTCF binding was observed upstream of alternatively spliced exons (34).

### Beyond the roadblock model

The ‘roadblock’ model describes the phenomenon of CTCF-mediated RNAPII elongation stalling downstream of included exons (33). However, this role does not seem applicable to CTCF binding sites that are highly enriched upstream of alternatively spliced exons (34). In addition, it also does not fully explain how CTCF occupancy exerts its effects in an upstream or downstream direction. Moreover, the optimal distance between a CTCF binding site and the proximal alternatively spliced exon where RNAPII accumulation occurs remains elusive.

The association of CTCF binding with RNAPII stalling at specific sites has been previously observed genome-wide in multiple cell lines (58), particularly at CTCF sites located immediately downstream to promoters of specific genes (59) including the p53 target genes *PUMA* and *p21* (35). Within these promoters, a higher RNAPII pausing index was associated with more proximal CTCF binding sites (59). Interestingly, CTCF-mediated RNAPII stalling was also detected at sites where CTCF binds upstream to promoters (59), which is consistent with Marina *et al.* (34). Given that RNAPII elongation is sterically hindered by CTCF bound at downstream regions, alternative mechanism(s) regulating CTCF-mediated RNAPII pausing at upstream sites could be involved. For instance, chromatin architecture influences splicing decisions via modulation of RNAPII elongation rate and recruitment of splicing factors (reviewed in (56), more details provided in *GENOMIC FEATURES* section).

### CTCF’s interplay with other factors

As a master regulator of gene expression, CTCF has the capacity to regulate the expression of numerous epigenetic modulators as well as genes involved in the transcriptional and splicing machinery. These include splicing factors, methylation regulators, chromatin remodelers, and histone modifiers (5,15,22,29). In regard to CTCF acting as a roadblock to AS of the archetypal *CD45* gene, CTCF has been directly linked to regulation of the three factors (RNAPII, DNMT-mediated methylation and TET-mediated demethylation) in different contexts (58,60–62). For example, besides its role in pausing RNAPII elongation, CTCF recruits and activates RNAPII when CTCF directly interacts with its largest subunit, POLR2A, via its C-terminal domain (58). In addition, CTCF also binds to and activates poly(ADP-ribose) polymerase (PARP1), which inactivates DNA methyltransferase DNMT1 and thereby inhibits DNA methylation at CTCF binding sites (60,61). Moreover, CTCF physically interacts with active TET enzymes and promotes DNA demethylation at specific sites (62). The upstream regulatory effects of CTCF on these proteins could add further complexity to the ‘roadblock’ model (33,34), particularly in the context of their possible recruitment at a genome-wide level. Therefore, the consequences of this regulatory network on modulating AS demands further experimental verification.

### The CTCF-PARP1 regulatory axis

An additional role for CTCF in modulating AS could be established from its known effect on activating PARP1 and its poly(ADP-ribosyl)ation (PARylation) activity (60,61). PARylation is a post-translational modification which is catalyzed by active PARP enzymes transferring ADP-ribose moieties from nicotinamide adenine dinucleotide (NAD^+^) to specific amino acid residues on the target substrate (63,64). It maintains various biological processes including genomic stability, chromatin architecture, transcriptional regulation, mitosis and cell death (64,65). While CTCF-regulated PARP1-PARylation activity has not been verified yet, inhibition of PARP1 expression or its PARylation activity has been linked to AS regulation. This has been demonstrated via PARP1-nucleosome interactions at target exon/intron boundaries or PARylation-mediated regulation of splicing factors including recruitment of spliceosomal factor 3B subunit 1 (SF3B1), a U2 snRNP (small nuclear ribonucleoprotein) spliceosomal member (66). A follow up study found that RNAPII elongation rate was also modulated at these sites depending on chromatin structure, which is disrupted upon PARP1 depletion (67). PARP1, in addition to its role as a PARylation activator, has been linked to regulation of transcription and AS via multiple mechanisms involving chromatin modulation, transcriptional modulation, post-transcriptional regulation of RNA-binding proteins and mRNA stabilization (reviewed in (68,69)). While this is true, possible roles for CTCF in the regulation of AS via activating PARP1 and PARylation will reveal new aspects of AS regulatory mechanisms, which require further investigation (Figure 2B).

Given its direct effect on PARP1 and its PARylation activity (60,61), CTCF might possibly regulate AS via RNA-binding proteins, whose binding, recruitment and expression are directly regulated by PARP1 and/or PARylation (68) (Figure 2B and C). This could be tested by examining the effect of CTCF on PARP1-mediated recruitment of SF3B1 and subsequently pre-mRNA splicing (66). Proteomics analysis of CTCF-interacting partners in MCF10A cells identified RNA-binding proteins including snRNPs, heterogeneous nuclear ribonucleoproteins (hnRNPs) and serine-arginine proteins (70), which are essential components of the splicing machinery (51). In addition, CTCF can transcriptionally regulate or directly interact with multiple splicing factors. For instance, the interaction between CTCF and POLR2A activates RNAPII (58), which provides a platform for factors regulating transcription initiation, elongation and termination as well as RNA processing (71). CTCF was also found to accumulate at specific RNAPII termination sites on protein-coding and small nuclear RNA (snRNA) genes (72).

A study focused on CTCF-RNAPII dynamics showed that CTCF regulated RNAPII elongation at the early elongation checkpoint of *c-myc*, and RNAPII termination at *U2* snRNA gene via regulating the recruitment or activation of key factors, such as DRB-sensitivity-inducing factor (DSIF) and negative elongation factor (NELF) (42). Moreover, CTCF interacts with and recruits positive transcription elongation factor-b (P-TEFb) which stimulates transcriptional elongation by phosphorylating POLR2A, NELF and DSIF (42). The working model at the *c-myc* site depends on CTCF binding downstream of the *c-myc* promoter and recruitment of DSIF to stall RNAPII elongation followed by P-TEFb recruitment which elicits continuing RNAPII elongation and RNA processing (42). Whether CTCF modulates AS at this and other genomic sites via this working model still awaits investigation. Together, the direct effect of CTCF on RNAPII elongation, PARP1/PARylation activity and splicing factor recruitment support the notion that CTCF can modulate AS via co-transcriptional regulation. Characterization of more splicing factors, methylation regulators and other AS factors directly or indirectly regulated by CTCF will contribute to our understanding of the complex mechanisms by which CTCF modulates AS.

## GENOMIC FEATURES

### A regulatory framework for splicing provided by CTCF-mediated chromatin architecture

**Figure 3.**
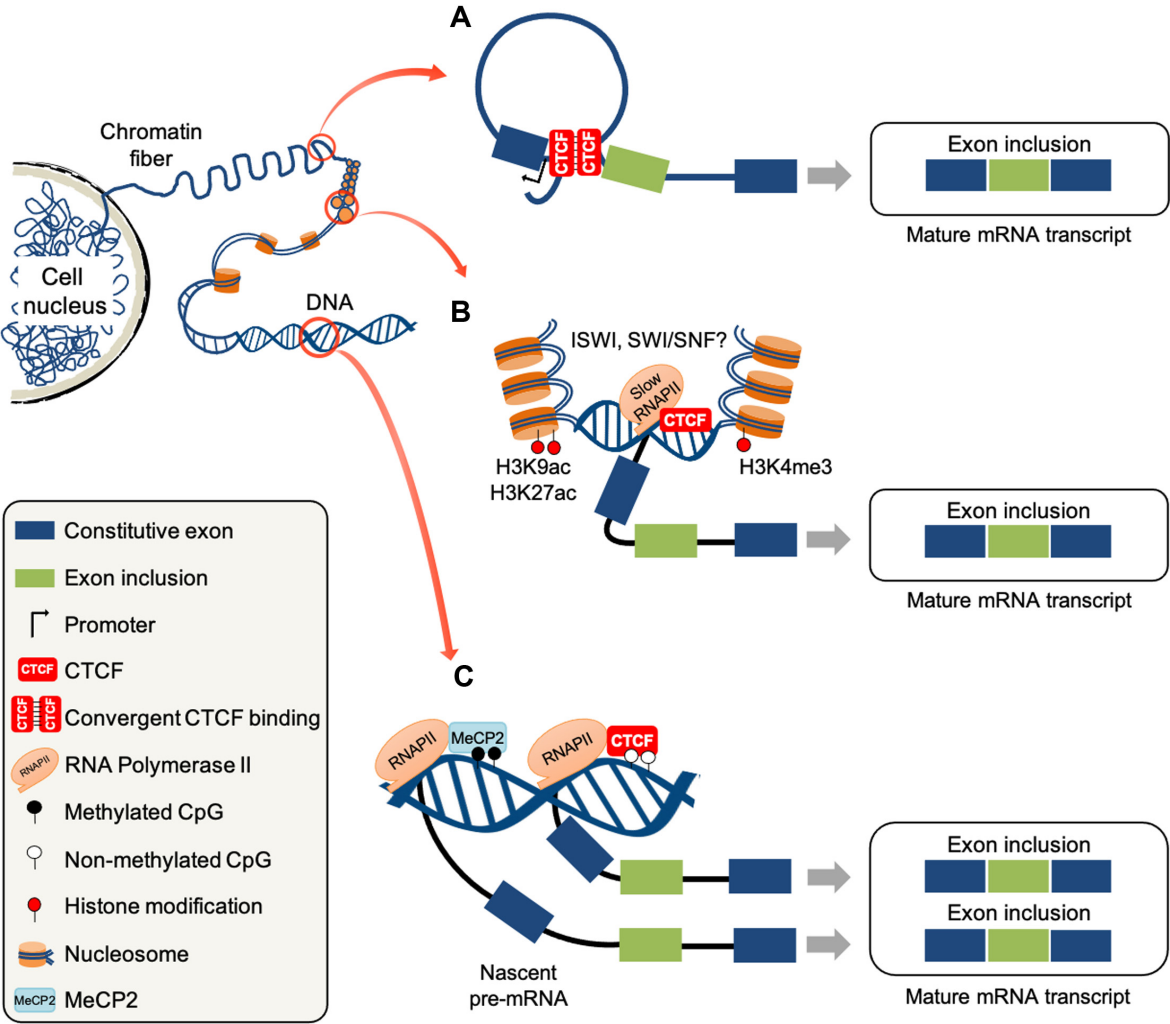
CTCF-mediated genomic and epigenetic regulation of alternative splicing. Schematic representation of putative genomic roles for CTCF in AS. (**A**) Formation of chromatin loops to bring alternatively spliced exons into physical proximity of the gene promoter (44). (**B**) Nucleosome occupancy-mediated altering of RNAPII elongation rate. (**C**) DNA methylation differentially regulates AS by CTCF and MeCP2. Unmethylated CpGs within CTCF sites promote exon inclusion via CTCF binding while DNA methylation favors exon inclusion via MeCP2 binding (38).

Intragenic CTCF binding sites, particularly those proximal to splice junctions, influence pre-mRNA splicing decisions, and thus mediate alternative exon or intron inclusion (33–38,40–42,44). Chromatin looping is a genome-wide function of CTCF placing it at the center of 3D chromatin architecture. Moreover, it brings genomic loci into spatial proximity allowing CTCF to modulate gene expression (30–32). The analysis of ChIP-seq, RNA-seq and high‐throughput chromosome conformation capture (Hi-C) data have revealed the widespread formation of CTCF-mediated chromatin loops between promotors and intragenic regions (44). Interestingly, chromatin loops formed upstream of exons induced their inclusion by bringing these alternatively spliced exons into physical proximity of the gene promoter (Figure 3A). This particular study provides a putative genome-wide link between intragenic CTCF-mediated chromatin looping and AS regulation (44).

Such a phenomenon affects members of the protocadherin *Pcdh* gene family, which encode multiple isoforms responsible for neuronal cell surface diversity (36,37). CTCF in conjunction with the cohesin complex mediates DNA looping between *Pcdh* enhancers and active isoform-specific promoters which induces alternatively spliced isoforms in a methylation-dependent manner (36,37). Mutation or methylation of CTCF sites at the promoter proximal regions abolishes CTCF binding *in vitro*. Furthermore, CTCF knockdown leads to a reduction in CTCF binding as well as the cohesin complex subunits RAD21 and SMC1 recruitment/binding and subsequently chromatin looping-mediated enhancer/promoter communications (36). The latter was also observed by RAD21 knockdown, which showed no effect on CTCF binding (36,37). Despite the direct link between CTCF/cohesin-mediated DNA looping and modulation of *Pcdh* isoforms, the exact underlying AS regulatory mechanism and downstream physiological consequences are yet to be elucidated.

RNAPII elongation is prone to stalling at specific CTCF/cohesin sites (35,40,73). This leaves open the possibility that CTCF might regulate AS via integrating chromatin looping and co-transcriptional factors. While CTCF-mediated RNAPII stalling has been verified (33–35,42), cohesin has been found to be recruited to sites where RNAPII pausing occurs, particularly when associated with CTCF (40,74). The CTCF/cohesin complex downstream of the *PUMA* and *p21* promoters has been shown to stall RNAPII elongation and regulate their expression (35). Similar to CTCF-mediated *Pcdh* isoform regulation (36,37), knockdown of CTCF abrogates CTCF binding at the *PUMA* and *p21* promoters and reduces cohesin complex (RAD21 and SMC1) levels (35). Likewise, CTCF-mediated cohesin recruitment and chromatin looping hinders RNAPII elongation downstream of proximal poly(A) sites in a methylation-dependent manner. This links CTCF to pre-mRNA alternative polyadenylation (40), a process allowing usage of alternative poly(A) sites to produce multiple mRNA transcripts from an individual gene (reviewed in (75)). Knowing that CTCF recruits cohesin to sites where RNAPII pausing occurs (35,40,73), it is possible that CTCF-mediated RNAPII elongation stalling induces cohesin recruitment and thereby chromatin looping, which leads to AS. Indeed, these findings highlight a new role of CTCF-mediated 3D chromatin organization in AS regulation. Conversely, CTCF-mediated AS might also itself be involved in shaping the genome.

### A role for CTCF-anchored nucleosomal occupancy

Nucleosome positioning is known to modulate RNAPII kinetics by acting as a physical barrier hindering RNAPII elongation and thus promoting the recognition of exon-intron boundaries by the splicing machinery (76). Nucleosomes are preferentially positioned in exons rather than introns or alternatively spliced exons, highlighting a role for nucleosome positioning in exon definition (77–80). Furthermore, nucleosome occupancy has been correlated with regulating AS, specifically exon inclusion (78–80) and intron retention (81). Exon-associated nucleosomes are enriched in active H3K36me3 and repressive H3K27me2 histone modifications, which help demarcate exon-intron boundaries (77,82). H3K36me3 is particularly enriched at actively transcribed genes (82) and associated with different splicing decisions involving recruitment of splicing regulators in a context-dependent manner (83,84).

Interestingly, several studies have reported an enrichment of CTCF sites around nucleosomes genome-wide, indicating that CTCF acts as an anchor for positioning nucleosomes in a symmetrical distribution (7,85–87). Yet, it is still unknown whether CTCF binds proximal to well-positioned nucleosomes or whether these nucleosomes are positioned depending on CTCF binding. SNF2H, the ATPase subunit of the ISWI chromatin remodeler, was found to be essential for global organization of nucleosomes and TAD formation via promoting CTCF binding. Knockdown of SNF2H causes a genome-wide reduction in CTCF binding but without affecting its abundance (88). Furthermore, CTCF was identified as an interacting partner of BRG1, the ATPase subunit of the chromatin remodeling complex SWI/SNF (89). While BRG1 depletion has no effect on genomic CTCF binding (88), it results in reduced nucleosome occupancy around CTCF sites (90). Moreover, knowing that PARylation mediates chromatin decondensation (65), CTCF-activated PARylation can possibly alter RNAPII dynamics via chromatin relaxation (Figure 2B). Taken together, it would be interesting to examine how CTCF and nucleosome positioning alters RNAPII elongation and subsequently AS (Figure 3B). Nucleosome positioning is perhaps the least studied mechanism linked to CTCF-mediated AS, which warrants further experimental investigation.

## EPIGENETIC FACTORS

### Impact of DNA methylation on CTCF occupancy

Constitutive splicing depends on many factors including the guanine and cytosine (GC) content, which is higher in exons compared to introns. DNA methylation contributes to the definition of exon-intron splice sites depending on the enrichment of CpG dinucleotides within the spliced exons/introns (91,92). It has been shown that 40% of variable CTCF occupancy is linked to differential CpG methylation, concentrated at two main positions within the canonical CTCF consensus sequence (93). Methylation of CpGs within CTCF target sites can prevent CTCF binding (94–98), which has been found to be correlated with DNA methylation patterns in a tissue-specific manner (93). The interplay between CTCF and DNA methylation in modulating AS has been described at a genome-wide or individual transcript level (36–41,43,45).

Genome-wide methylation profiling of multiple tissues could reveal an association between DNA methylation and alternatively spliced transcripts (43). This is interesting, given that CTCF binding sites often occur in GC-rich regions, typically in CpG islands (8,99). These transcripts were highly enriched in CTCF binding motifs and mutually exclusive exons in a position-dependent context (43). As an exemplar, CTCF was found to regulate a pair of mutually exclusive exons observed in synaptic calcium ion channel *Cacna1b* transcription, where CTCF binding to hypomethylated exon 37a (but not 37b) promotes its recognition and inclusion in neurons (41). Methylation of the exon 37a locus, on the other hand, leads to the inhibition of CTCF binding and exon 37b inclusion. The resulting Cacna1b isoform alters the calcium ion channel plasticity of the neuron (41). However, the mechanism by which CTCF modulates splicing at these sites has not been fully explored.

### Learning from *BDNF*: an exemplar of CTCF-regulated AS

CTCF is important for exon inclusion by pausing RNAPII-mediated transcription in a methylation-dependent manner (33,34). However, in one archetypal example, CTCF binding is required to protect against mis-splicing in brain-derived neurotrophic factor (*BDNF*), in a seemingly RNAPII elongation-independent manner (38). Mammalian *BDNF*, which is essential for regulating neurogenesis, synaptic plasticity, learning, memory and cognition, encodes multiple transcripts generated by AS (100,101). In turtle brain, intra-exonic splicing of *BDNF* causes a 40 bp deletion wholly within the protein coding exon IV, generating the functionally distinct BDNF2a isoform which has a 13 amino acid deletion and a truncated out-of-frame 21 amino acid C-terminus (102). When methyl-CpG-binding protein MeCP2 binds to its cognate site upstream of the *BDNF* intra-exonic splice site, it recruits the splicing factor Y-box binding protein (YB-1), and generates this truncated BDNF2a isoform. Interestingly, TET1 binding at MeCP2 sites was also essential for *BDNF* splicing, which is inhibited by knockdown of MeCP2, YB1 or TET1 (38).

Moreover, suppression of the TET1-MeCP2-YB1 complex, using a neural correlate of a classical conditioning process in turtles, induces site-specific DNA demethylation which primes CTCF binding to *BDNF*, proximal to the alternatively spliced locus. This interaction leads to shielding of canonical *BDNF* splicing and inhibition of its truncation, without RNAPII elongation stalling (38). The methylation status within the *BDNF* promoter region appears to govern the *BDNF* splicing decision. While methylated CpG dinucleotides provide docking sites for MeCP2 binding, DNA demethylation dissociates MeCP2 and promotes CTCF binding (38). Similarly, DNMT inhibition-mediated hypomethylation in turtle or intracellular NAD biosynthesis inhibition-regulated DNA hypermethylation in mouse, both modulate *BDNF* transcription via triggering dissociation of MeCP2 or CTCF, respectively, from the *BDNF* promoter region (39,103). In addition, NAD-depleted mouse cortical neurons have decreased cohesin recruitment and binding to the *BDNF* promoter, indicating a relationship between chromatin structure and *BDNF* transcription (39). Using hippocampal-specific depletion of CTCF in mice, CTCF/cohesin binding proximal to the *BDNF* promoter was also observed upon fear-conditioning. This attenuated their learning and memory activities as well as expression of key learning-related genes including *BDNF*, *Arc*, *Reln* and *Ppp1c* (104). Altogether, the interplay between CTCF and the TET1-MeCP2-YB1 complex in modulating *BDNF* splicing is influenced by chromatin structure and their functional impact remains to be elucidated.

### DNA methylation governs CTCF/MeCP2/TET1 interplay

Since MeCP2 is known for its role in regulating methylation-dependent AS (38,105,106), the link between CTCF and MeCP2 may help reveal an important underlying mechanism of CTCF-mediated AS. DNA methylation mediates opposing effects on the role of CTCF and MeCP2 binding to DNA and subsequently regulation of pre-mRNA splicing (Figure 3C). Apart from MeCP2/CTCF-mediated *BDNF* splicing (38), both MeCP2 and CTCF can promote exon inclusion in a context-dependent manner by binding to methylated or unmethylated DNA, respectively (33,34,105). Conversely, exon exclusion could occur via DNA demethylation or methylation inhibiting MeCP2 or CTCF binding, respectively. Both CTCF- and MeCP2-mediated exon inclusion have been linked to modulating RNAPII kinetics (33,34,105). While CTCF physically blocks RNAPII elongation (33,34), MeCP2-mediated pausing of RNAPII elongation is linked to recruiting histone deacetylase (HDAC) activity and inducing histone hypoacetylation (105), the inhibition of which increases the RNAPII elongation rate and subsequently exon exclusion (107). In addition, the DNA- and RNA-binding protein YB-1 physically interacts with both CTCF (108,109) and MeCP2 (38,110,111). While MeCP2-mediated recruitment of YB-1 has been linked to AS (38,111), the impact of CTCF and YB-1 interaction on pre-mRNA splicing has not been verified yet.

TET1 is another mutual interacting partner of CTCF (62) and MeCP2 (103,112), through which it regulates AS (33,34,38). Knowing that MeCP2 protects methylated 5mC from TET1-mediated oxidation (113), it is not very clear how abolishing MeCP2 binding to *BDNF* reduces TET1-catalyzed demethylation during the conditioning process, yet promotes CTCF binding (38). This could be explained by the notion that MeCP2 and TET1 are binding partners (103,112), implying that dissociation of MeCP2 possibly removes the whole TET1-MeCP2-YB1 complex. Alternatively, TET1 dissociation might be compensated by TET3, whose binding to *BDNF* has been found to increase following conditioning (38), and important for learning-dependent gene expression and behavioral adaptation (114). Given the common factors acting in CTCF- and MeCP2-mediated AS, their combined or independent roles on modulating AS are still poorly understood and require further studies particularly at a genome-wide level.

### Lessons from *CTCF* haploinsufficiency in human and mouse

While CTCF binding is inhibited by DNA methylation (94–98), CTCF itself regulates DNA methylation and preserves methylation-free regions throughout the genome (115–117). Moreover, CTCF knockdown induces global hypermethylation, particularly at CTCF binding sites, as well as loss of CTCF binding associated with altered expression of CTCF-regulated genes (118). Interestingly, two individuals with an intellectual disability and diagnosed with *CTCF* heterozygous deletions exhibited hypermethylation at nearly 300 CTCF binding sites genome-wide (119). The regulation of genome-wide methylation by CTCF was also demonstrated in *Ctcf* hemizygous mice, which are an ideal model of Ctcf haploinsufficiency. *Ctcf* haploinsufficient mice exhibit aberrant hypermethylation and increased spontaneous tumor formation within various organs (120). Using a similar model, we recently examined the effect of *Ctcf* haploinsufficiency on differential AS in several mouse tissues including brain, kidney, liver, muscle and spleen (45). Our analysis showed an overall perturbation of the AS landscape, causing mostly exon skipping, in a tissue-specific manner. This was despite Ctcf dosage only decreasing less than 40% in most tissues examined (45). We also detected a significant tissue-specific increase in intron retention events in *Ctcf* haploinsufficient liver and kidney, primarily affecting short introns with high GC content. The intron retention events in the liver showed that Ctcf-mediated intron retention has a differing impact on the mRNA expression of the intron-retaining genes. Interestingly, there was enrichment of Ctcf binding sites proximal, particularly upstream, to the majority of the differentially retained introns in mouse liver (45).

Knowing that *Ctcf* haploinsufficiency (119,120) or knockdown (118) induces hypermethylation, we proposed that *Ctcf* haploinsufficiency increased intron retention through a methylation-dependent mechanism (45), which may involve altering the kinetics of RNAPII elongation or chromatin looping. However, this hypothesis requires further direct experimental validation, considering possible indirect effects of decreased *Ctcf* levels. To account for these effects, it would be useful to examine the direct effect of CTCF depletion on global AS by using the auxin-inducible degron system. The degron system has been successfully used to study the acute and reversible effect of CTCF depletion on TAD boundaries and chromatin looping as well as gene expression (121–123). In addition, mutating CTCF binding sites using genome editing could provide another experimental approach to study CTCF-mediated AS at the individual transcript level. Given the cell-type-specific effects of CTCF on AS (45), further investigations into the specific mechanism are required.

### A chromatin code enriching AS regulation

Genome-wide chromatin-dependent regulation of AS involves association of CTCF, HP1α and histone modifications (46). The heterochromatin protein (HP1) family members (α, β and γ), which can co-localize with the heterochromatin-associated histone modification H3K9me3, have been shown to regulate the impact of DNA methylation on AS in a position-dependent manner. Binding of HP1 to an exon or its upstream intron was associated with diminishing or enhancing exon inclusion, respectively. This methylation-dependent binding modulates RNA splicing by recruiting splicing factors, such as SRSF3, to the methylated site (124). Yet, it remains to be determined how changing HP1 binding sites leads to different AS decisions. A proposed ‘chromatin code’ highlighted by enrichment of HP1α, CTCF, the transcriptional gene silencer Argonaute (AGO1), RNAPII and select histone marks around AS-regulated exons governs different modes of exon inclusion or skipping (46). The interaction of RNAPII with AGO1 (125) affects its elongation in the context of HP1α-mediated chromatin condensation thus causing higher inclusion of alternative exons (126). Knowing that HP1α recruits and interacts with CTCF (127), it would be reasonable to postulate that AS can result from crosstalk between HP1 and CTCF. In addition, given the association between CTCF and HP1α proximal to included exons (46), it would be interesting to further explore whether CTCF binding contributes to altering exon splicing decisions. This might be facilitated by DNA methylation and HP1-mediated splicing factor recruitment (124) (Figure 2C). Alternatively, CTCF may modulate AS by slowing RNAPII elongation via HP1α-mediated chromatin condensation at methylated sites (128).

The distribution of CTCF binding sites coincides with nucleosome positioning (7,85–87). These studies demonstrated that enrichment of CTCF binding sites is detected around nucleosomes associated with histone variants H2A.Z and H3.3 and multiple histone modifications. The co-localization of CTCF with these histone variants and modifications marks the boundaries of histone methylation domains and was linked to chromatin-mediated gene expression regulation (7,85–87). Consistent with the above, CTCF binding along with active histone modifications H3K9ac, H3K27ac or H3K4me3 have been shown to be highly enriched in ‘more-included’ exons compared to ‘less-included’ exons (129). However, correlations between the repressive histone modifications H3K27me3 and H3K9me2 and CTCF binding sites have been detected around alternatively skipped exons in MCF7 more than MCF10A cells (46). For example, the *PUMA* locus harbors an intragenic chromatin boundary consisting of the active marks H3K4me3 and H3K9ac and the repressive mark H3K9me3, important for CTCF-mediated regulation of *PUMA* (35). Interestingly, CTCF knockdown reduces H3K9me3 levels within *PUMA* as well as at *p21* loci (35). Moreover, a broad putative role for CTCF in regulating diverse histone modifications could be achieved via PARP1 and PARylation (65). These findings argue for a plausible link between CTCF-associated histone modifications and pre-mRNA splicing outcome, which might involve regulation of RNAPII kinetics and/or splicing factor recruitment via chromatin-binding proteins.

## CONCLUDING REMARKS AND FUTURE DIRECTIONS

Transcriptomic diversity is governed by AS, which is orchestrated by complex regulatory mechanisms comprising genomic, epigenetic, and transcriptional layers (51,130). The versatile and ubiquitous factor CTCF plays diverse roles in these regulatory layers through multimodal mechanisms. However, there are still gaps in our understanding of the interplay between these mechanisms. Further research is required to gain a clearer picture of the CTCF-mediated AS regulatory network. Combined computational and experimental efforts are required to foster a comprehensive mechanistic understanding of the experimentally verified and putative AS regulatory roles mediated by CTCF (Table 1). Overall, most evidence of CTCF-mediated AS regulation has been found at the individual transcript level while the putative roles are mostly described at a genome-wide level.

**Table 1.** Summary of the known mechanisms of CTCF in modulating alternative splicing. CTCF-mediated AS regulatory mechanisms are divided into verified or putative roles

Mechanism	Context	Model	Description	Biological relevance	Ref
**Verified mechanisms**					
RNAPII elongation*	*CD45*	Human Burkitt lymphoma B cells	CTCF binding to *CD45* exon 5 promotes exon inclusion via stalling RNAPII elongation	Lymphocyte development	(33,34)
RNAPII elongation	*PUMA, p21*	Human HCT116 colorectal carcinoma cells	CTCF binding recruits cohesin downstream to *PUMA* and *p21* promoters, which stalls RNAPII elongation and regulates their expression	p53-mediated apoptotic response	(35)
DNA methylation	*BDNF*	Turtle brain tissue, mouse embryonic cortical neurons	CTCF binding to unmethylated target sites proximal to *BDNF* promotes canonical *BDNF* splicing	Learning-dependent activities	(38,39)
DNA methylation	*Cacna1b*	F11 (rat dorsal-root ganglion neurons/mouse neuroblastoma hybrid) cells	CTCF binding to hypomethylated sites at *Cacna1b* promotes mutually exclusive exons	Calcium ion channel plasticity	(41)
Chromatin architecture*	*Pcdh*	Human SK-N-SH and mouse N2a neuroblastoma cells, mouse CAD catecholaminergic neuronal tumor cells	CTCF/cohesin-mediated DNA looping induces alternatively spliced *Pcdh* isoforms	Neuronal cell surface diversity	(36,37)
Chromatin architecture*	Multiple transcripts	Human HCT116 colorectal carcinoma cells	CTCF/cohesin-mediated DNA looping induces alternative polyadenylation	Altering cancer transcriptome	(40)
Splicing factor recruitment	*c-myc, U2 snRNA*	HeLa cells	CTCF controls RNAPII elongation and termination via recruitment of NELF, DSIF and P-TEFb	Gene expression	(42)
**Putative mechanisms**					
RNAPII elongation	Genome-wide	Human Burkitt lymphoma B cells	Association between CTCF and RNAPII elongation proximal to included exons	-	(33,34)
RNAPII elongation	Genome-wide	Various cells	Association between CTCF binding and RNAPII stalling at specific sites	-	(58,59)
DNA methylation	Genome-wide	Mouse retina and brain tissues	Association of CTCF binding sites and DNA methylation in alternatively spliced transcripts	-	(43)
Chromatin architecture*	Genome-wide	Human lymphoblastoid cells	CTCF-mediated chromatin loops bring alternatively spliced exons into physical proximity of gene promoter	-	(44)
DNA methylation	Genome-wide	Mouse brain, kidney, liver, muscle and spleen tissues	*Ctcf* haploinsufficiency mediates tissue-specific changes in AS events, notably an increase in intron retention in *Ctcf* haploinsufficient liver and kidney	-	(45)
Histone modification	Genome-wide	Various cells	Association between CTCF binding and histone modifications close to alternatively spliced exons	-	(35,46,129)
Miscellaneous	Genome-wide	Human MCF7 breast cancer cells Non-tumorigenic MCF10A mammary cells	Association between CTCF and HP1α proximal to included exons which may involve splicing factor recruitment	-	(46)
Miscellaneous*	Genome-wide	Various cells	CTCF-mediated activation of PARP1 and PARylation govern AS via hypomethylation of CTCF binding sites, RNAPII elongation, chromatin relaxation and splicing factor recruitment	-	(60,61,66–69)

*denotes involvement of methylation-regulated CTCF sites.

Enrichment of CTCF binding sites proximal to alternatively spliced exons has been linked to methylation, RNAPII elongation, chromatin looping, histone modifications and splicing factor recruitment (33–46). To date, most studies have examined RNAPII elongation- and DNA methylation-related mechanisms. However, other CTCF-mediated mechanisms regulating AS, particularly nucleosome positioning, chromatin architecture and histone modifications, remain largely uncharacterized. While CTCF appears to spatio-temporally coordinate AS, it is still unknown whether these factors have cooperative, competitive, dependent, or independent relationships in CTCF-mediated AS. In addition, the extent of CTCF’s role in modulating AS remains uncertain in regard to its specificity to individual transcripts versus genome-wide levels, ubiquitous versus cell-type-specific CTCF target sites and cellular versus systemic functions. It is worth noting that the association of CTCF-mediated AS events and resulting biological functions have not been extensively studied. Some studies have already shed light on several functions influenced by this role such as lymphocyte development (33,34), p53-mediated apoptotic response (35), learning-dependent activities (38), neuronal cell surface diversity (36), calcium ion channel plasticity (41) and the impact of *Ctcf* haploinsufficiency (45).

Pursuing these avenues of research present many computational challenges including the complexities of analyzing the transcriptome and epigenome as well as current limitations of the available bioinformatic tools in detecting AS events, annotating protein isoforms and providing optimal data resolution and coverage (reviewed in (131,132)). There are several data resources, such as ENCODE (encodeproject.org), the Cancer Genome Atlas (TCGA: https://www.cancer.gov/tcga), and the Cancer Cell Line Encyclopedia (portals.broadinstitute.org/ccle), which are useful for comprehensive analysis of AS landscapes in various normal and malignant cells. Given frequent allelic variations and somatic mutations in CTCF (133–136), it would be important to establish links between aberrant CTCF-mediated AS and cancer. Knowing that CTCF and its testis-specific paralogue CTCF-like (CTCFL)/BORIS have some mutual genomic occupancy (137), a new role for BORIS in AS regulation may also warrant further investigation. Comprehensive data analyses should aim to resolve the reciprocal and mutually exclusive interplay between CTCF and key factors involved in AS regulation rather than studying CTCF-mediated effects on an individual factor.

Structural changes in the genome arising from genetic variation or introduced experimentally via deletion or inversion of CTCF sites using genome editing can have pathological consequences. Disrupting the highly conserved architecture of TADs has been shown to have a dramatic impact on development and normal spatio-temporal control of gene expression (138–140). While the impact of genetic variation on CTCF-mediated chromatin looping has been well established (138–140), the consequences for the AS landscape is only now being revealed. Allelic variation at CTCF sites adjacent to exons can both affect CTCF binding and exon inclusion (44). Future analyses will seek to explore correlations between transcriptomic and genomic data and cellular or developmental phenotypes. Moreover, given the extensive characterization of CTCF as a tumor suppressor gene (120,136,141–143), its frequent mutation in cancer (133,134,136) and loss- or gain-of-function phenotypes (135), causal links between CTCF-mediated aberrant AS and cancer are likely to emerge (144,145).

To date, the role of CTCF in AS has been mostly elucidated using computational approaches, while experimental validation remains challenging especially at a genome-wide level. This is further complicated by the fact that CTCF is essential for somatic cell viability (143), genome architecture (30,32) and gene expression regulation (5,22). Integrating the known roles for CTCF in regulating chromatin architecture and gene expression with AS-mediated effects on transcriptome and proteome diversity would provide opportunities for future directions. For instance, it is still unknown how AS is impacted by CTCF-mediated TADs and compartmentalization of chromatin. In addition, CTCF is known to transcriptionally regulate many key AS-regulating genes and interact directly with proteins and RNA molecules leading to direct or indirect effects on AS. Ultimately, unravelling the underlying mechanisms by which CTCF regulates AS may address the question as to what determines the fates of CTCF-mediated alternatively spliced transcripts and their biological functions.
